# Editorial: Harnessing bacteriophages and phage-engineered products for antibacterial and anticancer therapies: challenges and opportunities

**DOI:** 10.3389/fmicb.2026.1799584

**Published:** 2026-03-03

**Authors:** Steve Petrovski, Tamás Fehér, Swapnil Ganesh Sanmukh

**Affiliations:** 1 Department of Microbiology, Anatomy, Physiology and Pharmacology, La Trobe University, Bundoora, VIC, Australia; 2 La Trobe Institute of Molecular Sciences, La Trobe University, Bundoora, VIC, Australia; 3 Institute of Biochemistry, HUN-REN Biological Research Centre, Szeged, Hungary; 4 Université Clermont Auvergne, iGReD, CNRS UMR 6293, INSERM U1103, Clermont-Ferrand, France; 5 Centre de Recherche en Nutrition Humaine d'Auvergne, Clermont-Ferrand, France; 6 Groupe Cancer Clermont Auvergne, Clermont-Ferrand, France

**Keywords:** endolysin, One Health, phage engineering, phage enzymes, phage therapy

Bacteriophages (phages) are viruses that infect and kill bacteria and represent the most abundant biological entities on Earth. They play essential roles across microbial ecology, biotechnology, and human health. Understanding phages is crucial for revealing how bacterial populations are regulated in natural ecosystems, where phage-driven predation shapes microbial community structure, influences nutrient cycling, and maintains ecological balance. In medicine, phages have re-emerged as powerful alternatives or complements to antibiotics, offering highly targeted therapies that eradicate pathogenic bacteria while sparing the commensal microbiota, an increasingly valuable advantage as antimicrobial resistance continues to rise globally. Beyond their therapeutic potential, phages have become indispensable tools in molecular biology and biotechnology, enabling innovations such as gene-editing platforms, high-throughput ligand discovery, and sensitive bacterial detection technologies. As research further uncovers the diversity, mechanisms, and engineering potential of phages and phage-derived enzymes, these viruses are increasingly recognized as versatile natural agents capable of deepening our understanding of microbial life, guiding the development of next-generation antimicrobials, and providing novel strategies for combating infectious disease.

This Research Topic highlights the expanding promise of phage biology across antibacterial and anticancer applications. As antibiotic resistance escalates, phages and their engineered derivatives are gaining renewed attention as precise, adaptable bio-nanoparticles capable of targeting pathogenic bacteria while preserving beneficial microbiota. Advances in phage biology, virus-phage hybrids, and engineered phage components, including capsids, tail fibers, peptides, and lytic enzymes, have broadened the scope of their medical and industrial applications. Despite this progress, important questions remain regarding therapeutic efficacy, safety, manufacturability, and regulatory approval, underscoring the need for continued multidisciplinary investigation.

## Contributions

This Research Topic brings together four review articles and seven original research articles that explore phage mechanisms of action, engineered phage-based products, and their potential in both antibacterial and anticancer therapies. Collectively, these contributions aim to stimulate discussion, advance translational research, and illuminate the opportunities and challenges shaping the future of phage-based innovation.

## Review Article

Across four comprehensive reviews, Canning et al., Singh et al., Wang et al., and Castellanos et al. illustrate the breadth and dynamism of current phage research across clinical, environmental, and oncological contexts.

Canning et al.'s *Phage therapy to treat cystic fibrosis Burkholderia cepacia complex lung infections* underscores the profound therapeutic potential of phages against notoriously difficult-to-treat BCC infections. Their review highlights key translational barriers, including the dominance of temperate phages, narrow host ranges, and the absence of streamlined pipelines for rapid phage matching and manufacturing.

Complementing this clinical focus, Singh et al.'s mini-review *Phage therapy for environmental biotechnology applications* positions phages as sustainable, One Health-aligned biocontrol agents across agriculture, industrial fermentation, and aquatic systems. However, they also emphasize persistent challenges, such as GMP bottlenecks, regulatory uncertainty, and host-range limitations, which currently restrict widescale adoption.

Wang et al., in *Advancements in research leveraging phage display technology for gastric cancer diagnosis and treatment*, broaden the conceptual landscape by illustrating how phage display accelerates biomarker discovery, targeted imaging, and ligand-guided therapeutics in oncology. Their review highlights the impact of next-generation sequencing, library engineering, and AI-driven selection strategies, while acknowledging limitations such as tumor heterogeneity and reduced post-translational modification.

Meanwhile, Castellanos et al.'s *Endolysins: targeting Streptococcus pneumoniae in its major anatomical niches of infection* shifts the focus from whole phages to phage-derived enzymes. They detail how modular endolysins, including Pal, Cpl-1, and Cpl-711, enable rapid, resistance-sparing killing of *S. pneumoniae* across the lungs, middle ear, bloodstream, and CNS. Their analysis underscores the promise of engineered chimeric lysins while identifying critical needs for improved delivery platforms, pharmacokinetic optimisation, and regulatory alignment.

Together, these four reviews provide a panoramic overview of how phage-based platforms, from virions to engineered enzymes and display systems, are rapidly evolving to meet challenges spanning environmental sustainability, cancer diagnostics and therapy, and difficult respiratory infections.

## Research articles

Across seven original research articles, phage biology and phage-derived antimicrobials emerge as powerful and rapidly advancing tools for combating antimicrobial resistance, enhancing biotechnological processes, and expanding diagnostic and therapeutic capabilities.

Li et al. analyze the novel *Shewanella xiamenensis* phage SXP01, demonstrating how highly stable, lytic phages with modular endolysins can deliver sustainable pathogen control in aquaculture settings, supported by strong *in vivo* protection of fish and detailed genomic characterization.

Shi et al. characterize the broad-host-range *Pseudomonas aeruginosa* phage Pae01, showcasing its potent activity against multidrug-resistant clinical isolates and its ability to disrupt biofilms alone or synergistically with gentamicin, findings with important implications for managing chronic and device-associated infections.

Addressing a critical translational challenge, Parmar et al. evaluate the Biolog Omnilog and Agilent Cytation platforms for phage susceptibility testing. Their results provide the strongest evidence to date that these high-throughput liquid-based techniques are both reproducible and scalable, paving the way for personalized phage therapy, yet call our attention to the current lack of their standardization.

At the molecular level, Chu et al. advance phage-derived enzyme therapeutics by characterizing LysKP213, a remarkably thermostable endolysin from a *Klebsiella pneumoniae* phage. Their work demonstrates how pairing lysins with outer-membrane permeabilisers or antimicrobial peptides can unlock potent antibacterial activity against Gram-negative pathogens.

Gorodnichev et al. deliver one of the most comprehensive assessments of phage-antibiotic interactions in *K. pneumoniae*, testing multiple phages, antibiotics, and a depolymerase across several strains. Their findings reveal strong synergy in many cases, but also strain-specific and method-dependent outcomes, including occasional antagonism. The importance of choosing the appropriate analytical process to assess potential interactions is also highlighted.

Ruizhe et al. contribute another therapeutically promising phage, vB_Aba_QH4, a highly virulent *Acinetobacter baumannii* phage with impressive stability, rapid adsorption, and significant protective effects in invertebrate infection models. Its genomic safety profile further reinforces its translational potential.

Finally, Kizheva et al. expand the global phage repertoire by isolating and characterizing new *Enterococcus faecalis* phages from wastewater, including the novel species vB_SEF_8. Their work demonstrates strong lytic activity against antibiotic-resistant strains, broad pH and temperature tolerance, and efficacy in a milk-based model, valuable for both clinical and food-safety applications.

## Conclusion

Together, the contributions in this Research Topic underscore the remarkable versatility and accelerating momentum of phage science. From whole-phage therapeutics to engineered enzymes, display technologies, and advanced susceptibility testing platforms, these studies highlight both the current maturity and the future potential of phage-based solutions across medicine, biotechnology, agriculture, and environmental systems. As antimicrobial resistance continues to rise and the need for novel therapeutic and diagnostic strategies becomes increasingly urgent, bacteriophages and phage-engineered products are poised to play a central role in next-generation biomedical innovation. The collective insights presented here deepen our understanding of phage biology and illuminate the translational pathways and technological advances that will shape the future of phage-driven antibacterial and anticancer therapies. They also underline future needs of the field, e.g., for standardized assessment of phage therapeutic potential, for well-defined regulatory pipelines to translate laboratory findings to clinical applications, or for the exhaustive analysis of the ecological impact of therapeutic applications in veterinary settings. We hope this Research Topic fosters continued interdisciplinary collaboration and accelerates progress toward real-world applications that harness the full potential of these extraordinary biological agents.

